# Beyond Tobacco: Bridging Gaps in Social History Records for Tobacco‐Free Nicotine Pouch Consumers

**DOI:** 10.1002/oto2.70034

**Published:** 2024-11-12

**Authors:** Taylor J. Stack, Morgan N. McCain, Ezer H. Benaim, Theresa A. Dickerson, Ibtisam Mohammad, Brent A. Senior, Adam J. Kimple, Christine DeMason

**Affiliations:** ^1^ Department of Otolaryngology–Head and Neck Surgery University of North Carolina at Chapel Hill Chapel Hill North Carolina USA

**Keywords:** cigarette, oral cancer, synthetic nicotine, vape pen, vaping

## Abstract

**Objective:**

Nicotine‐containing products have historically been tobacco derivatives like cigarettes, cigars, and dip. Recently, tobacco‐free nicotine (TFN) products have been marketed as a healthy alternative. TFN pouches are small, discreet, flavored pouches containing nicotine designed to be placed between the gum and lip. This product does not fit a conventional tobacco category, leading to inaccurate reporting. This study aims to investigate discrepancies in physician documentation of TFN pouches.

**Methods:**

A retrospective chart review was conducted on TFN users.

**Setting:**

Single Health Care System.

**Methods:**

Statistical analyses assessed TFN documentation concordance between social history templates and physician notes.

**Results:**

There were 150 patients who used TFN and 841 patients who vaped. Concordance was higher for vape documentation than TFN pouch documentation (55.9%, 470/841 vs 25.3%, 38/150; *P* < .001). Of those who used TFN, 60% (90/150) were classified as “Smokeless Tobacco Users” in the social history; however, 35 were inaccurately classified as chew, and 17 did not specify TFN use. Only 38 specified TFN use; only 25% (38/150) of records demonstrated concordance.

**Conclusion:**

Only 25% of records were concordant with physician notes, highlighting the need for a designated place for TFN use within social history templates. Nicotine use history is crucial in the setting of microvascular reconstruction and cosmetic surgeries. Thus, accurate reporting is crucial for future research on the long‐term effects of TFN. This study's findings underscore a deficit in current social history templates and the need to recognize TFN pouches as distinct entities.

While vaping, cigarette, and e‐cigarette use rates are declining,^1^, ^2^ nicotine pouches are rising in popularity. In 2016, tobacco‐free nicotine (TFN) pouches entered the US market, and sales have increased exponentially since their arrival (Figure 1).^3^, ^4^ What was once only available as a tobacco‐derived substance now exists in many synthetic forms and flavors. While the negative impacts of smokeless tobacco, such as chew or snuff, are well described,^5^, ^6^, ^7^, ^8^, ^9^ and detrimental effects of vaping are emerging, little is known about the impact of TFN pouches. These new products do not fit into most social history templates of electronic health records (EHRs), and many physicians are unaware of them due to their novelty.^10^

**Figure 1:**
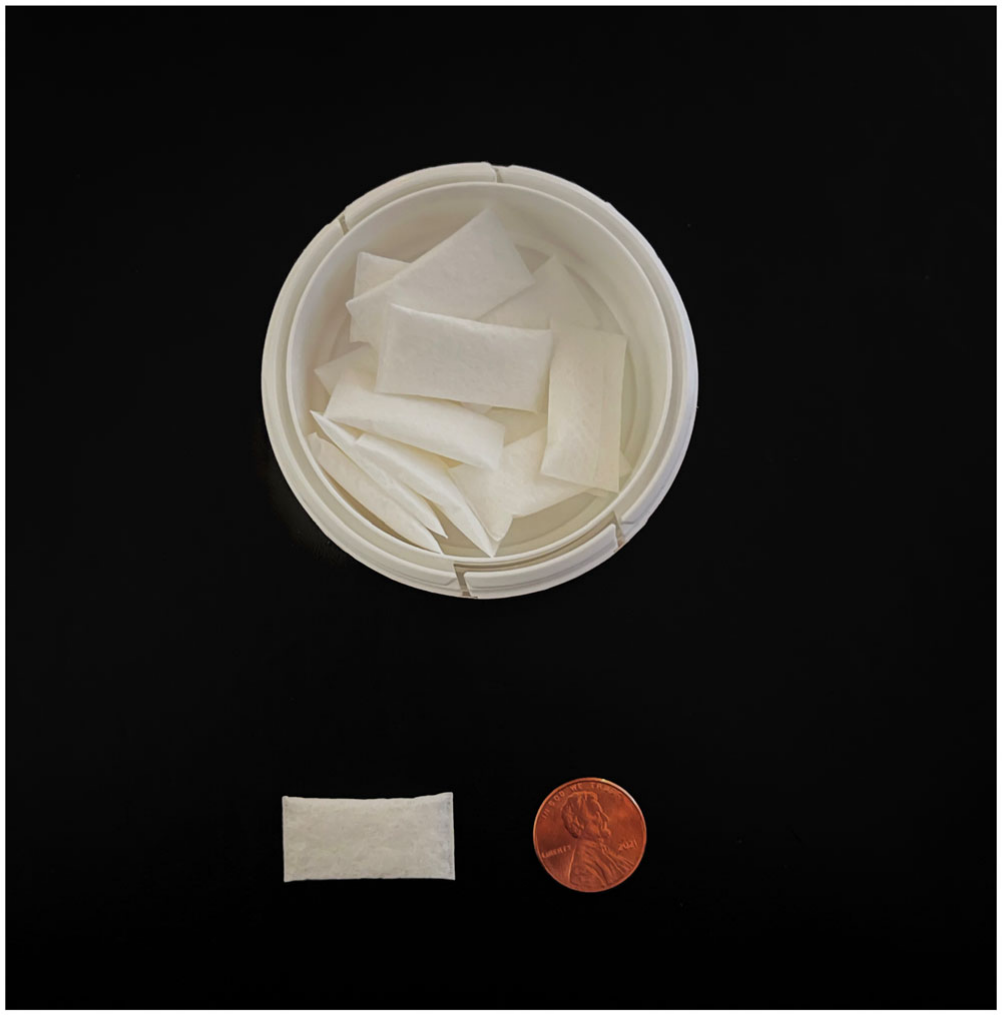
A tobacco‐free nicotine (TFN) pouch with a penny for size reference. Most TFN pouches are 28‐32 mm in diameter.

With over 228 flavors,^11^, ^12^ TFN pouch sales have rapidly increased since they were first marketed in 2016. In a 2022 survey, 9.8% of 942 young adults reported using nicotine pouches.^13^ According to the 2023 National Youth Tobacco Survey, e‐cigarettes were the most used product (7.7%) among middle and high schoolers, followed by cigarettes (1.6%), cigars (1.6%), and TFN pouches (1.5%).^2^ While e‐cigarettes remain the most‐used tobacco product, sales peaked in 2019 and have steadily declined since 2020.^1^, ^14^ Among high schoolers, e‐cigarette use declined from 14.1% to 10.0% between 2022 and 2023.^2^

In contrast with the decline of e‐cigarette use, TFN pouch sales have increased considerably from 2016 to 2022.^3^, ^4^ Sales for 2022 were 808.14 million units, a considerable increase from 126.06 million units sold in 2019.^4^ The public perception of these products is unique compared to other tobacco products.^15^ While no manufacturer has US Food and Drug Administration approval to market TFN pouches as a tobacco cessation product, these products are marketed as a smoke‐free, spit‐free tobacco alternative that can be used anywhere.^16^ Yet, a survey of 2464 young adults found that most misunderstood what “tobacco‐free nicotine” means, shedding light on this confusing marketing tactic.^17^ Although TFN pouches contain a similar nicotine content to tobacco‐derived nicotine,^18^ their use is perceived as less harmful than tobacco‐derived nicotine products, and many view these TFN pouches as “cleaner” and associate them with “high class.”^15^, ^19^, ^20^, ^21^ Although perceived as a healthier alternative to tobacco, studies have found that those who use both TFN pouches use them in addition to other tobacco products.^22^, ^23^

The risks associated with traditional smokeless tobacco (chew, dip) are well‐described.^24^, ^25^ Emerging data has elucidated proinflammatory cytokine effects and mucosal changes associated with tobacco‐derived nicotine pouches.^25^, ^26^ Specifically, smokeless tobacco keratosis (STK) has been described as caused by chronic irritation. STK is the most common lesion found in those who use smokeless tobacco, developing in 47% of chronic users.^27^ Similar to tobacco‐derived nicotine, chronic TFN pouch use causes systemic and local toxicological responses.^11^ Vaping synthetic nicotine has been linked to lung cancer,^28^ but data on the long‐term effects of TFN pouches does not currently exist. Given that nicotine promotes vasoconstriction and thrombosis and affects vascular health and wound healing,^5^, ^6^, ^7^, ^29^ TFN pouch use may impact surgical outcomes, making accurate reporting crucial for optimizing patient care.

This scenario is all too familiar, as e‐cigarettes and vape pens entered the market in the early 2010s. The emergence of this product changed social history questioning, as “Do you smoke?” no longer sufficed. A 2017 quality improvement study found that most physicians screened for tobacco, but very few screened for vape use. This study showed that the most effective way to increase screening was to add a vape‐specific question to the EHR template, increasing vape screening from 0% to 78%.^30^ Now, most EHR social history templates have questions about smokeless tobacco, e‐cigarette, and vape use; however, there is no straightforward way to document TFN pouch use.

The failure to recognize TFN pouch consumption during medical evaluations, including preoperative assessments, can have implications for treatment decisions and surgical outcomes.^5^, ^6^, ^7^ Given the novelty of these products, accurate documentation is necessary to understand the associated health risks. Our study aims to investigate the accuracy of nicotine pouch reporting within health care documentation systems. Specifically, we seek to evaluate the adequacy of current EHR templates in capturing nicotine pouch use and compare this against what physicians document in their clinical notes. By elucidating discrepancies and identifying potential gaps in documentation practices, we encourage refining health care protocols and ensuring a comprehensive and accurate assessment of nicotine exposure among patients.

## Methods

This retrospective, cross‐sectional review evaluated the accuracy of nicotine product documentation in patient notes and social history templates. This study aimed to compare TFN pouch documentation with vape documentation, which has a designated checkbox in the social history template. Patient notes were queried using the Electronic Medical Record Search Engine (EMERSE). A variety of specialty notes were reviewed for this study, including primary care and ENT. The timeframe was from 2015 to 2023. Clinical note documentation was compared to the clinical “snapshot,” which contains a social history template. If the social history template was consistent with what was recorded in the physician's note, the record was considered “concordant.”

The record was considered concordant if a patient was checked as a former or current smokeless tobacco user, and TFN pouch use was specified in the comments box. On the contrary, if a physician's note stated that the patient used a TFN pouch, but “snuff” was checked in the social history template, this was considered discordant. Similarly, the records were discordant if the social history was blank or stated that the patient did not use any tobacco products. If a patient who vaped had a check by the “vape” box in the social history template, the record was considered concordant. This study was approved by the Office of Human Research Ethics at UNC Institutional Review Board #23‐2680.

### Sample Selection

The EMERSE operates similarly to Google in retrieving medical text information from clinical notes and orders.^31^ EMERSE contains integrated data from the University of North Carolina (UNC) Health's EHR system, Epic (Epic Systems Corporation). EMERSE can be used to search through clinical documents of 4.4 million patients within the UNC health system. To identify patients who use TFN pouches, EMERSE was queried for the terms “nicotine pouch,” “Zyn pouch,” “TFN pouch,” and “nicotine‐free pouch.” Of note, “Zyn” is a popular TFN pouch brand name. This search resulted in 150 patients who had matching TFN terms in their clinical notes. To identify the records of those who vape, EMERSE was queried for the terms “continues to vape,” “continuing to vape,” “vaping,” and “vape‐pen.” Because “vape” and “electronic cigarettes” are options in the social history template, queries using these words had outputs of millions of records, many of which were false positives due to this terminology being present in most charts. Because it was not attainable to filter through these records, a sample of 841 was gathered using the terms stated above.

### Social Vulnerability Index (SVI)

The SVI is based on 15 social variables, including poverty, household composition, minority status and language, and housing and transportation. The aggregate score offers granular data at the US county and census tract‐level, and is calculated using home addresses. Because previous studies show associations between socioeconomic status and tobacco use, proximity to stores that sell tobacco, and likelihood of tobacco cessation, this study utilized the 2020 Centers for Disease Control and Prevention SVI as a baseline covariate to evaluate associations between social determinants of health and documentation accuracy.^14^, ^32^, ^33^, ^34^ The SVI value of census tracts ranges from 0.000 to 1.000, with 1.000 representing extreme social vulnerability and 0.000 representing the lowest level of social vulnerability.

### Statistical Analysis

Data normality was assessed using the Shapiro‐Wilk test. Pearson's *χ* ^2^ test, Wilcoxon rank sum test, and Fisher's exact test were used to compare the vape and TFN pouch groups. Medians and interquartile ranges (IQRs) were calculated. Multivariate logistic regression assessed associations between sex, age, and SVI with record concordance. All analyses were performed using RStudio (Version 2023.06.0 + 421, RStudio Inc).

## Results

**Figure 2:**
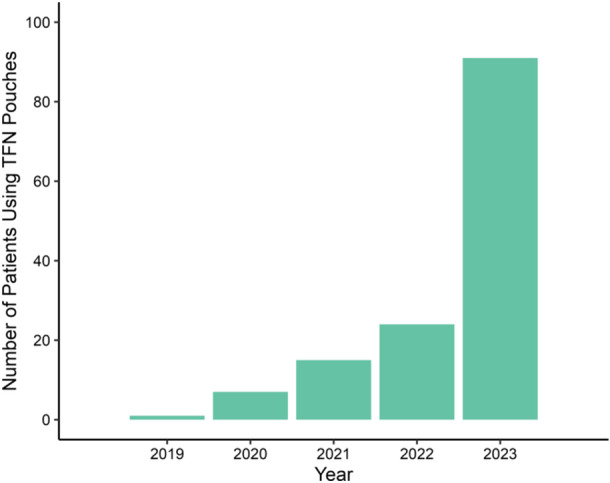
The number of patients using tobacco‐free nicotine (TFN) pouches increased from 2019 to 2023 (*η* ^2^ = 0.46), as identified in the local electronic health record through Electronic Medical Record Search Engine.

There were 150 records with TFN pouch use documentation and 841 with vape use documentation. The 2 groups differed in baseline covariates, including age and sex (Table 1). For those who used TFN pouches, 8.0% (12/150) were female, whereas for those who vaped, 51.5% (433/841) were female (*P* < .001). Those who used TFN pouches had a younger median age than those who vaped (36.9 years, IQR 27.1‐52.5, vs 45.0 years, IQR 31‐59; *P* < .001). From 2019 to 2023, there was a substantial increase in the number of patients who used TFN pouches from 1 to 91 (Figure 2).

**Table 1:** Baseline Covariates of Those Who Use TFN Pouches and Vape

Characteristic	TFN pouch, N = 150^a^	Vape, N = 841^a^	*P* value^b^
Sex			<.001
Female	12 (8.0%)	433 (51.5%)	
Male	138 (92.0%)	408 (48.5%)	
Age, y	36.9 (27.1, 52.5)	45.0 (31.0, 59.0)	<.001
Race			.10
American Indian/Alaskan native	1 (0.7%)	20 (2.4%)	
American Indian/Alaskan native and white	0 (0.0%)	1 (0.1%)	
Asian	0 (0.0%)	6 (0.7%)	
Asian and white	0 (0.0%)	2 (0.2%)	
Black/African American	5 (3.3%)	63 (7.5%)	
Black/African American and white	0 (0.0%)	2 (0.2%)	
Latine	0 (0.0%)	1 (0.1%)	
Native Hawaiian or other Pacific Islander	0 (0.0%)	1 (0.1%)	
Other	2 (1.3%)	26 (3.1%)	
Unknown	4 (2.7%)	4 (0.5%)	
White	138 (92.0%)	715 (85.0%)	
SVI	0.37 (0.2, 0.7)	0.52 (0.3, 0.8)	<.001
Concordance	38 (25.3%)	470 (55.9%)	<.001

Abbreviations: IQR, interquartile range; SVI, Social Vulnerability Index; TFN, tobacco‐free nicotine.

^a^ n (%); median (IQR).

^b^ Pearson's *χ* ^2^ test; Wilcoxon rank sum test; Fisher's exact test.

Most of the TFN pouch records had documentation inconsistencies or errors. Out of 150 of those who use TFN pouches, 90 were classified as “Smokeless Tobacco Users” in the social history. However, 35 of these were inaccurately classified as using chew or snuff, and 17 did not specify TFN pouch use. The remaining 38 specified TFN pouch use; therefore, only 25.3% (38/150) of records demonstrated concordance between physician notes and social history templates. Sixty‐four of those who used TFN pouches also had a cigarette smoking history, of which 98% (63/64) were concordant. In summary, in the same group of patients, cigarette documentation, which has a checkbox in the social history template, displayed higher concordance than TFN pouch (63/64 vs 38/150; *P* < .001). 

Concordance was higher for vape documentation compared to TFN pouch documentation (55.9%, 470/841 vs 25.3%, 38/150; *P* < .001). Vape documentation had 2.3 times the odds of being concordant than TFN pouch (95% confidence interval = 1.58‐3.47). Even after age and sex matching the groups, TFN pouch documentation remained less concordant than vape documentation (38/150, 25.3% vs 88/150, 58.7%; *P* < .001) (Supplemental Table S1, available online). Those who vaped had higher median SVIs than those who used TFN pouches (0.52, IQR 0.2‐0.7, vs 0.34, IQR 0.3‐0.8, *P* < .001). In multivariate logistic regression, SVI was not associated with concordant documentation in either group.

## Discussion

With the evolution of nicotine consumption, it is crucial to understand the patterns of usage and potential health risks associated with TFN pouches. This study shows discrepancies between physician notes and EHR social history templates, making it challenging to track TFN pouches usage accurately.

Despite TFN pouches gaining popularity, the concordant documentation of their usage within EHR systems lags behind vaping (25.3% vs 55.9%; *P* < .001), which has a designated place in social history templates. Our data indicate a gap in health care documentation of TFN pouches, which may be attributed to inadequate EHR documentation templates and a lack of physician awareness. In a previous study, adding a vape‐specific question to EHR templates was the most effective way to increase screening.^30^ Therefore, the adaptation of the EHR template to include a nicotine pouch‐specific question would likely improve documentation and physician awareness of TFN use.

Consistent with other studies, those using TFN pouches were relatively young (median age: 36.9 years) and predominantly male (138/150, 92.0%).^14^, ^22^ Previous studies show associations between socioeconomic status and increased likelihood of engaging in smokeless tobacco use.^34^ Areas with lower median household incomes tend to have a higher number of stores that sell tobacco products.^33^, ^34^ Those from lower socioeconomic groups are more likely to use tobacco products and less likely to successfully quit smoking, even after accessing cessation programs.^14^, ^32^

Considering this, SVI was compared among those who used TFN pouches and vape pens. SVI was higher, correlating with higher social vulnerability, for those who vaped compared to those who used TFN pouches (0.52, IQR 0.2‐0.7, vs 0.34, IQR 0.3‐0.8, *P* < .001). Although SVI did not show a statistically significant association with concordant documentation in either group, understanding the socioeconomic factors influencing nicotine product usage and documentation practices is crucial for designing effective health care policies and interventions.

The risks of TFN pouches are largely unknown, as opposed to the negative health consequences of tobacco and nicotine. Nicotine has been shown to promote oxidative inflammation, thrombosis, angiogenesis, and vasoconstriction.^29^ Smokeless tobacco is associated with oral cancer, leukoedema, STK, periodontal disease, and submucosal fibrosis.^24^, ^25^ In addition to being a significant contributor to head and neck cancer,^8^, ^9^ general tobacco use is associated with less favorable outcomes in microvascular surgery.^5^, ^6^, ^7^ Cutaneous necrosis, surgical site infection, and flap failure have all been associated with tobacco use.^6^ These tobacco‐related complications extend beyond the surgical site, increasing the risk of intensive care unit admission, pulmonary complications, and neurological complications.^7^ Considering the known risks of tobacco use and nicotine in general, preoperative recognition of TFN pouch use may be important for patient safety and future investigations.

The study's limitations include its retrospective design and reliance on the EMERSE query, which may be subject to documentation and search errors. More specificially, our vaping cohort is a relatively small sample compared to national percentages due to the nature of our EMERSE query. This study population is also limited to 1 health care group and region. TFN pouches may be underreported in medical records due to their novelty and lack of a clear category within existing documentation templates, which could further bias the results. Future research should explore the long‐term health effects of TFN pouches, address the discrepancies in documentation practices, and develop standardized protocols for accurate documentation of emerging nicotine products within health care settings.

In conclusion, this study underscores the need for the adaptation of EHR templates and awareness of TFN pouches among health care professionals. Accurate documentation and physician education about TFN pouch use are crucial for comprehensive patient care, risk assessment, and the development of evidence‐based interventions to address the evolving landscape of nicotine consumption. By identifying gaps in documentation practices and addressing barriers to accurate reporting, health care systems will be better equipped to advise surgical patients and track health outcomes over time.

## Author Contributions

**Taylor J. Stack**, data collection, data analysis, manuscript preparation; **Morgan N. McCain**, manuscript preparation; **Ezer H. Benaim**, data collection, manuscript preparation; **Theresa A. Dickerson**, data analysis, manuscript preparation; **Ibtisam Mohammad**, study design, manuscript preparation; **Brent A. Senior**, manuscript revision; **Adam J. Kimple**, manuscript revision; **Christine DeMason**, project oversight, manuscript preparation.

## Disclosures

### Competing interests

No authors have any conflict of interest to disclose.

### Funding source

Research in this publication was supported by the National Institute on Deafness and Other Communication Disorders branch of the National Institute of Health (NIH) under award number T32DC005360 (E.H.B).

## Supporting information

Supporting information.
