# Supplementary material for: Beyond Tobacco: Bridging Gaps in Social History Records for Tobacco‐Free Nicotine Pouch Consumers
Source: OTO Open. 2024 Nov 12;8(4):e70034. doi: 10.1002/oto2.70034 (PMC11555441; doi:10.1002/oto2.70034)
Supplement: Supplementary file 1 — Supporting information. [file OTO2-8-e70034-s001.docx]

Supplemental Table 1. Baseline covariates of those who use tobacco-free nicotine (TFN) pouches and vape in the age and sex-matched cohort. Abbreviations: SVI, Social Vulnerability Index.

| **Characteristic** | **TFN**, N = 150*^1^* | **vape**, N = 150*^1^* | **p-value***^2^* |
| --- | --- | --- | --- |
| Sex |  |  | >0.9 |
| Female | 12.0 (8.0%) | 12.0 (8.0%) |  |
| Male | 138.0 (92.0%) | 138.0 (92.0%) |  |
| Age (years) | 36.9 (27.1, 52.5) | 37.0 (27.0, 52.8) | >0.9 |
| Concordance | 38.0 (25.3%) | 88.0 (58.7%) | <0.001 |
| Race |  |  | 0.2 |
| American Indian/Alaskan Native | 1.0 (0.7%) | 3.0 (2.0%) |  |
| Asian | 0.0 (0.0%) | 1.0 (0.7%) |  |
| Black/African American | 5.0 (3.3%) | 10.0 (6.7%) |  |
| Latine | 0.0 (0.0%) | 1.0 (0.7%) |  |
| Other | 2.0 (1.3%) | 7.0 (4.7%) |  |
| Unknown | 4.0 (2.7%) | 3.0 (2.0%) |  |
| White | 138.0 (92.0%) | 125.0 (83.3%) |  |
| *^1^* n (%); Median (IQR) | | | |
| *^2^* Pearson’s Chi-squared test; Wilcoxon rank sum test; Fisher’s exact test | | | |
